# Association of Anti-TNF Therapy with Ventricular Repolarization Indices in Patients with Rheumatoid Arthritis: A Longitudinal Electrocardiographic Study

**DOI:** 10.3390/jcm15207913

**Published:** 2026-10-13

**Authors:** Melih Pamukcu, Tugba Izci Duran, Bora Demircelik

**Affiliations:** 1 Department of Rheumatology, Health Sciences University, Etlik City Hospital, 06110 Ankara, Türkiye; 2 Clinic of Rheumatology, Republic of Türkiye Ministry of Health Denizli State Hospital, 20050 Denizli, Türkiye; 3 Clinic of Cardiology, Ankara Guven Hospital, 06540 Ankara, Türkiye

**Keywords:** rheumatoid arthritis, tumor necrosis factor inhibitors, electrocardiography, ventricular repolarization, arrhythmias

## Abstract

**Background:** Patients with rheumatoid arthritis (RA) have an increased risk of cardiovascular morbidity that cannot be fully explained by traditional cardiovascular risk factors alone. Chronic systemic inflammation has been implicated in myocardial electrical remodeling, yet the effect of anti-tumor necrosis factor (anti-TNF) therapy on ventricular repolarization remains insufficiently characterized. This study investigated longitudinal changes in electrocardiographic markers of ventricular repolarization following anti-TNF therapy in patients with RA. **Methods:** This retrospective longitudinal study included 115 patients with RA who initiated anti-TNF therapy and had clinical, laboratory, and 12-lead electrocardiographic assessments at baseline and after approximately 24 weeks. Ventricular repolarization was evaluated using QT, corrected QT (QTc), Tpeak–Tend (Tp-e), corrected Tp-e (cTp-e), and Tp-e/QT. Cross-sectional associations between repolarization indices and inflammatory/disease-related variables were assessed at 24 weeks, together with longitudinal correlations between changes in these variables and changes in repolarization indices. **Results:** The mean age was 54.4 ± 12.2 years, and 81.7% of participants were women. After 24 weeks of treatment, significant reductions were observed in CRP, ESR, DAS28-CRP, and HAQ scores (all *p* < 0.001). The Tp-e interval decreased from 85.1 ± 13.6 to 75.3 ± 13.5 ms, and the Tp-e/QT ratio decreased from 0.20 ± 0.04 to 0.18 ± 0.03 (both *p* < 0.001), with moderate within-subject effect sizes for both changes (Cohen’s *dz* = 0.46). At 24 weeks, Tp-e and Tp-e/QT were positively correlated with inflammatory markers, disease activity, functional impairment, and disease duration. However, longitudinal change-to-change analyses showed no consistent associations between changes in inflammatory/disease activity measures and changes in repolarization indices, except for a weak correlation between ΔDAS28-CRP and ΔTp-e (*r* = 0.201, *p* = 0.031). Despite a significant reduction in mean QTc, the proportion of patients with prolonged QTc remained unchanged (18.3% at both assessments; McNemar *p* = 1.00). **Conclusions:** Anti-TNF therapy in patients with RA was associated with significant improvements in several ECG-derived ventricular repolarization indices alongside reductions in inflammatory and disease activity. However, the lack of consistent longitudinal correlations and the unchanged prevalence of prolonged QTc indicate that these findings should be interpreted as treatment-associated changes rather than evidence of a direct electrophysiological or antiarrhythmic effect of TNF inhibition. Prospective controlled studies incorporating clinical cardiovascular outcomes are warranted.

## 1. Introduction

Persistent immune-mediated inflammation primarily affecting the synovial joints leads to progressive structural damage, functional impairment, and a wide range of systemic manifestations, a clinical entity known as rheumatoid arthritis (RA). Among its systemic complications, cardiovascular disease remains one of the leading causes of excess mortality despite substantial advances in disease-modifying therapies [1,2]. Despite advances in disease-modifying therapies, patients with RA continue to experience a substantially higher incidence of cardiovascular complications than the general population, reflecting the combined influence of conventional cardiovascular risk factors and chronic systemic inflammation [3,4].

The mechanisms responsible for the increased arrhythmic burden in RA are multifactorial. In addition to ischemic heart disease, chronic inflammation may directly alter myocardial structure and electrophysiology by promoting fibrosis, impairing electrical conduction, and increasing ventricular repolarization heterogeneity. These alterations facilitate the development of ventricular arrhythmias through reduced repolarization reserve and enhanced susceptibility to re-entry mechanisms [5,6]. Tumor necrosis factor-α (TNF-α) has a pivotal role in both RA pathogenesis and myocardial remodeling, providing a biological rationale for investigating the potential cardiovascular effects of TNF inhibition [7].

Electrocardiographic (ECG) indices reflecting ventricular repolarization have emerged as practical, non-invasive tools for assessing arrhythmic susceptibility [8]. Among the available ECG-derived indices, the Tpeak–Tend (Tp-e) interval and the Tp-e/QT ratio provide indirect measures of ventricular repolarization heterogeneity and have been linked to an increased susceptibility to ventricular arrhythmias across diverse cardiovascular conditions [9]. Previous investigations have demonstrated impaired ventricular repolarization in patients with RA; however, evidence regarding the influence of anti-TNF therapy on these ECG-derived parameters remains scarce.

Given the central role of TNF-α in both systemic inflammation and myocardial remodeling, we hypothesized that effective suppression of inflammatory activity may be accompanied by favorable changes in ventricular repolarization. Accordingly, this study explored whether suppression of TNF-mediated inflammation is associated with changes in ECG-derived ventricular repolarization indices, particularly the Tp-e interval and Tp-e/QT ratio, in patients with rheumatoid arthritis.

## 2. Materials and Method

### 2.1. Patient Selection

This retrospective longitudinal observational study included 115 consecutive patients with RA who fulfilled the 1987 revised American College of Rheumatology (ACR) classification criteria for RA and had initiated anti-TNF therapy between January 2021 and December 2024 [10]. Following approval of the study protocol by the Local Ethics Committee, eligible patients were retrospectively identified from the institutional records. Available clinical, laboratory, and 12-lead ECG data obtained at anti-TNF initiation (baseline) and approximately 24 weeks after treatment initiation were retrieved and analyzed. Written informed consent for participation was obtained from all patients included in the study. The study was conducted in accordance with the Declaration of Helsinki.

In accordance with the Turkish Social Security Institution reimbursement criteria applicable during the study period, anti-TNF therapy was initiated in patients with active RA who had an inadequate response despite treatment with at least three different conventional synthetic disease-modifying antirheumatic drugs (csDMARDs), including methotrexate, administered at appropriate doses for at least three months each, and who had persistent moderate-to-high disease activity. The anti-TNF agents used in the study were adalimumab (40 mg subcutaneously every other week), etanercept (50 mg subcutaneously once weekly), and golimumab (50 mg subcutaneously once monthly). All patients included in the study received anti-TNF therapy in combination with methotrexate and were evaluated after approximately 24 weeks of treatment. All patients received concomitant methotrexate at a dose of 15–25 mg/week, administered orally and/or subcutaneously, together with folic acid supplementation 5 mg/week. Methotrexate treatment had been initiated before anti-TNF therapy and was continued throughout the 24-week observation period. The methotrexate dose and route of administration remained stable during follow-up. Other concomitant medications with potential effects on ventricular repolarization were also required to remain unchanged during the observation period. Patients who required initiation, discontinuation, or dose modification of medications known to affect ventricular repolarization during follow-up were excluded. Patients receiving systemic corticosteroids or csDMARDs other than methotrexate were also excluded.

To minimize potential cardiovascular and metabolic confounding, patients were excluded if they had elevated cardiac biomarkers (troponin or CK-MB), known coronary artery disease, diabetes mellitus, hypertension, left ventricular ejection fraction < 50%, cardiomyopathy, clinically significant valvular heart disease, cardiac conduction abnormalities, anemia, electrolyte disturbances, hypercholesterolemia, thyroid dysfunction, renal or hepatic dysfunction, pulmonary disease, or ECG recordings of insufficient quality for reliable repolarization measurements. Laboratory abnormalities used as exclusion criteria were defined as follows: anemia was defined as a hemoglobin level < 13 g/dL in men and <12 g/dL in women; serum potassium abnormalities were defined as potassium levels < 3.5 or >5.1 mmol/L; renal dysfunction was defined as an estimated glomerular filtration rate (eGFR) < 60 mL/min/1.73 m^2^; hepatic dysfunction was defined as aspartate aminotransferase (AST) or alanine aminotransferase (ALT) levels above the institutional upper limit of normal; and thyroid dysfunction was defined as a thyroid-stimulating hormone (TSH) level outside the institutional laboratory reference range. Patients meeting any of these criteria were excluded from the study. Patients receiving hydroxychloroquine, antiarrhythmic agents, tricyclic antidepressants, antihistamines, or antipsychotics were also excluded because of their potential effects on cardiac electrophysiology. Only patients in sinus rhythm at both baseline and 24-week ECG assessments were included in the analysis. As part of the standard pre-biologic evaluation, NT-proBNP was assessed to exclude patients with evidence suggestive of overt or subclinical heart failure.

Laboratory assessments included erythrocyte sedimentation rate (ESR) and C-reactive protein (CRP, mg/L). Serological status was assessed using rheumatoid factor (RF) and anti-cyclic citrullinated peptide (anti-CCP) antibodies, both recorded as positive or negative. Disease activity was assessed using the Disease Activity Score in 28 joints based on CRP (DAS28-CRP) [11] while functional status was evaluated using the Health Assessment Questionnaire (HAQ) [12].

### 2.2. Electrocardiographic Measurements

Standard 12-lead electrocardiograms were recorded before treatment initiation and repeated after approximately 24 weeks to determine ventricular repolarization parameters, including the QT interval, corrected QT (QTc), Tpeak–Tend (Tp-e) interval, and Tp-e/QT ratio.

#### 2.2.1. Tp-e Measurement and QTc Calculation

Standard 12-lead ECG recordings were obtained in the supine position using the same electrocardiograph (Cardiofax V, model 9320; Nihon Kohden, Tokyo, Japan) at a paper speed of 25 mm/s and a calibration of 10 mm/mV. Identical recording conditions were used at baseline and at the 24-week assessment. Clinical cardiovascular outcomes, including arrhythmias, cardiovascular events, cardiovascular hospitalizations, and sudden cardiac events, were not systematically assessed during the 24-week follow-up period.

ECG measurements were independently performed by two cardiologists who were blinded to patient identity, clinical and laboratory data, anti-TNF therapy information, and the temporal sequence of the ECG recordings (baseline vs. 24 weeks). Measurements from three consecutive cardiac cycles were obtained and averaged to minimize measurement variability.

#### 2.2.2. QT and QTc Measurement

The QT interval was measured from the onset of the QRS complex to the end of the T wave. QTc was calculated using Bazett’s formula (QTc = QT/√RR), where QT and RR intervals were expressed in seconds. The corrected Tp-e interval (cTp-e) was calculated using the corresponding heart-rate correction (cTp-e = Tp-e/√RR) [13,14,15]. Prolonged QTc was defined as QTc > 450 ms in men and >460 ms in women. A QTc ≥ 500 ms was additionally evaluated as a markedly prolonged QTc threshold.

#### 2.2.3. Tp-e Interval Measurement

The Tp-e interval was defined as the interval from the peak of the T wave to the end of the T wave. The end of the T wave was determined as the intersection of the tangent to the descending limb of the T wave with the isoelectric line. Tp-e measurements were obtained from the precordial leads, and measurements from three consecutive cardiac cycles were averaged [16,17].

#### 2.2.4. Ratio Calculations

Tp-e interval and Tp-e/QT ratio have been proposed as surrogate ECG markers of ventricular repolarization heterogeneity and have been associated with ventricular arrhythmias and adverse cardiovascular outcomes in previous studies [18,19].

The 24-week follow-up time point was selected to assess ventricular repolarization after a sustained period of anti-TNF therapy, allowing sufficient time for stabilization of the clinical and inflammatory response. ECG assessment was predefined at baseline and 24 weeks as part of the study protocol. An intermediate 12-week ECG assessment was not included in the protocol; therefore, the study was designed to evaluate medium-term pre–post changes rather than the temporal trajectory of repolarization during treatment.

### 2.3. Statistical Analysis

All statistical analyses were carried out using IBM SPSS Statistics for Windows (version 23.0; IBM Corp., Armonk, NY, USA). Continuous variables are reported as mean ± standard deviation (SD) or median with interquartile range (IQR), depending on data distribution, whereas categorical variables are expressed as counts and percentages. Data normality was evaluated using the Shapiro–Wilk test in conjunction with visual inspection of histograms and Q–Q plots.

Because baseline and 24-week measurements represented paired observations from the same patients, changes in continuous variables were evaluated using the paired-samples *t*-test for normally distributed paired differences and the Wilcoxon signed-rank test for non-normally distributed paired differences. Paired categorical variables were compared using McNemar’s test, where appropriate. Spearman’s rank correlation analysis was used to evaluate associations between ventricular repolarization indices and clinical and inflammatory continuous variables. Cross-sectional correlations were assessed using measurements obtained at the 24-week assessment. In additional longitudinal analyses, changes in inflammatory and disease activity measures were correlated with changes in ECG parameters. Changes (Δ) were calculated as the 24-week value minus the corresponding baseline value.

The proportions of patients with prolonged QTc (>450 ms in men and >460 ms in women) and markedly prolonged QTc (≥500 ms) were determined at baseline and at 24 weeks. Within-patient changes in QTc-threshold status were assessed using McNemar’s test. Effect sizes for paired continuous comparisons were calculated using Cohen’s *dz*, defined as the mean of the paired differences divided by the standard deviation of the paired differences. All statistical tests were two-sided, and a *p* value < 0.05 was considered statistically significant.

## 3. Results

A total of 115 patients with RA were included in the study, of whom 94 (81.7%) were women and 21 (18.3%) were men. The mean age was 54.4 ± 12.2 years, and the mean disease duration was 72.0 ± 11.2 months. Regarding anti-TNF therapy, 60 patients (52.2%) received adalimumab, 30 (26.1%) received etanercept, and 25 (21.7%) received golimumab. An overview of the participants’ baseline demographic, clinical, and laboratory data are provided in Table 1.

Following 24 weeks of anti-TNF therapy, significant improvements were observed in inflammatory markers and disease activity. Serum CRP and ESR decreased markedly (both *p* < 0.001), accompanied by significant reductions in DAS28-CRP and HAQ scores (both *p* < 0.001). Hemoglobin levels increased significantly after treatment (*p* < 0.001), whereas leukocyte, neutrophil, lymphocyte, and platelet counts declined significantly.

Heart rate and QRS duration remained unchanged throughout follow-up (*p* > 0.05). In contrast, significant reductions were observed in ventricular repolarization parameters after anti-TNF therapy. The Tp-e interval decreased from 85.1 ± 13.6 ms at baseline to 75.3 ± 13.5 ms at 24 weeks (*p* < 0.001). QTc and cTp-e intervals and the Tp-e/QT ratio also decreased significantly after treatment (all *p* < 0.001). The complete pre- and post-treatment ECG findings are presented in Table 2.

At baseline, 21 of 115 patients (18.3%) had a prolonged QTc, defined as >450 ms in men and >460 ms in women. At 24 weeks, 21 patients (18.3%) met the same criterion. Two patients moved from above to below the threshold, whereas two patients moved from below to above the threshold; therefore, there was no significant change in prolonged-QTc status (McNemar *p* = 1.00).

A markedly prolonged QTc (≥500 ms) was present in 3 of 115 patients (2.6%) both at baseline and at 24 weeks, with no significant change in paired QTc-threshold status (McNemar *p* = 1.00).

Effect-size analysis demonstrated moderate within-subject effects for the reductions in the Tp-e interval (Cohen’s dz = 0.46) and Tp-e/QT ratio (dz = 0.46), whereas the effect size for the change in QT interval was small (dz = 0.09). At the 24-week assessment, the Tp-e interval was positively correlated with CRP (r = 0.56, *p* < 0.001), ESR (r = 0.57, *p* < 0.001), DAS28-CRP (r = 0.29, *p* = 0.002), HAQ (r = 0.33, *p* < 0.001), and disease duration (r = 0.58, *p* < 0.001). Similarly, the Tp-e/QT ratio showed significant positive correlations with CRP (r = 0.43, *p* < 0.001), ESR (r = 0.53, *p* < 0.001), DAS28-CRP (r = 0.25, *p* = 0.007), HAQ (r = 0.22, *p* = 0.018), and disease duration (r = 0.49, *p* < 0.001) (Table 3).

In longitudinal change-to-change analyses, changes in CRP and ESR were not significantly correlated with changes in either Tp-e or Tp-e/QT. A weak positive correlation was observed between the change in DAS28-CRP and the change in Tp-e (r = 0.201, *p* = 0.031), whereas no significant correlation was observed between changes in DAS28-CRP and Tp-e/QT (r = 0.099, *p* = 0.291). Changes in HAQ were not significantly correlated with changes in either Tp-e or Tp-e/QT (Table 4).

## 4. Discussion

In this retrospective longitudinal study, anti-TNF therapy was associated with significant improvements in ECG-derived indices of ventricular repolarization in patients with RA. Following approximately 24 weeks of treatment, Tp-e interval, cTp-e, QTc interval, and Tp-e/QT ratio were all significantly reduced, accompanied by marked improvements in inflammatory markers, disease activity, and functional status. At the 24-week assessment, Tp-e and Tp-e/QT were positively correlated with CRP, ESR, DAS28-CRP, HAQ, and disease duration, supporting an association between inflammatory/disease burden and ventricular repolarization abnormalities. However, longitudinal change-to-change analyses did not demonstrate a consistent relationship between the magnitude of improvement in inflammatory or disease activity measures and changes in repolarization indices. Only the change in DAS28-CRP showed a weak correlation with the change in Tp-e. In addition, despite the reduction in mean QTc, the proportions of patients exceeding clinically relevant QTc thresholds did not change significantly. Taken together, these findings suggest that improvement in inflammatory activity and ventricular repolarization occurred in parallel during anti-TNF therapy, but do not establish a direct or proportional relationship between these changes.

Cardiovascular disease has become one of the major determinants of long-term prognosis in rheumatoid arthritis, accounting for a substantial proportion of excess mortality despite considerable therapeutic advances [20]. Although accelerated atherosclerosis has traditionally been considered the principal mechanism underlying cardiovascular complications in RA, contemporary evidence suggests that chronic immune-mediated inflammation also promotes myocardial electrical and structural remodeling independent of ischemic heart disease [2]. Persistent exposure to pro-inflammatory cytokines—including TNF-α, interleukin (IL)-1β, and IL-6—may induce oxidative stress, impair calcium handling, alter ion channel function, disrupt gap junction integrity, and promote myocardial fibrosis, thereby increasing ventricular repolarization heterogeneity and susceptibility to malignant ventricular arrhythmias [21]. This evolving concept has contributed to the emergence of cardio-rheumatology, which emphasizes the close interaction between systemic inflammation and cardiovascular disease in patients with immune-mediated inflammatory disorders.

TNF-α is not only a key mediator of synovial inflammation but also an important regulator of myocardial electrophysiological remodeling. Experimental studies have demonstrated that TNF-α signaling modifies cardiac ion channel expression, prolongs action potential duration, and promotes myocardial fibrosis, thereby facilitating ventricular electrical instability [21]. Consequently, suppression of TNF-mediated inflammation could plausibly be associated with changes in ventricular repolarization through multiple complementary mechanisms, including attenuation of inflammatory signaling, reduction in oxidative stress, preservation of gap junction function, and limitation of myocardial fibrotic remodeling. Nevertheless, our longitudinal change-to-change analyses are important in this context. Although inflammatory markers, disease activity, and several repolarization indices improved over the same treatment period, reductions in CRP and ESR were not significantly correlated with changes in Tp-e or Tp-e/QT. The only significant longitudinal association was a weak correlation between ΔDAS28-CRP and ΔTp-e. Therefore, the present findings should not be interpreted as demonstrating that the magnitude of reduction in systemic inflammation directly determines the magnitude of improvement in ventricular repolarization.

Previous studies have demonstrated ventricular repolarization abnormalities in patients with RA [22,23,24]. Aladag et al. reported prolonged Tp-e, Tp-e/QT, and Tp-e/QTc ratios, supporting the presence of subclinical ventricular electrical alterations in this population [23]. Our findings extend these observations by demonstrating longitudinal improvements in repolarization indices following anti-TNF therapy. These changes occurred in parallel with reductions in CRP, ESR, DAS28-CRP, and HAQ scores. However, the absence of consistent correlations between changes in inflammatory/disease activity measures and changes in repolarization indices suggests that these parallel improvements should not be interpreted as evidence of a direct relationship. Beyond its well-established efficacy in controlling joint inflammation, anti-TNF therapy has been associated with favorable cardiovascular effects in patients with rheumatoid arthritis [25,26,27]. This interpretation is biologically plausible, as systemic inflammation, oxidative stress, endothelial dysfunction, and adverse myocardial remodeling have been implicated in cardiovascular involvement in RA [2,20]. Nevertheless, in the absence of an untreated RA comparator group, the observed changes cannot be attributed specifically to TNF inhibition.

The clinical significance of ECG-derived ventricular repolarization indices remains an area of active investigation. Although the Tp-e interval and Tp-e/QT ratio are widely accepted as surrogate markers of ventricular repolarization heterogeneity, they should not be interpreted as direct measures of arrhythmic risk [9,28]. Associations between prolonged Tp-e-related parameters and ventricular tachyarrhythmias, sudden cardiac death, and adverse cardiovascular outcomes have been reported in several clinical settings [29,30,31,32]. However, these associations do not establish the prognostic significance of changes in these indices in patients with RA [23,24]. Importantly, although mean QTc decreased significantly in the present study, the proportion of patients with prolonged QTc (>450 ms in men and >460 ms in women) remained unchanged at 18.3%, and the proportion with QTc ≥ 500 ms remained unchanged at 2.6%. Thus, the statistically significant reduction in mean QTc did not translate into a detectable change in categorical QTc-risk thresholds over 24 weeks. These findings reinforce the need to interpret the observed ECG changes as changes in surrogate repolarization indices rather than evidence of reduced arrhythmic or cardiovascular events.

This study has several strengths. To our knowledge, it is among the few longitudinal studies evaluating changes in ventricular repolarization parameters before and after anti-TNF therapy in patients with RA. The paired longitudinal design allowed each patient to serve as their own control, reducing the influence of between-patient variability. The simultaneous assessment of inflammatory markers, disease activity, functional status, and multiple ventricular repolarization indices enabled both cross-sectional and longitudinal evaluation of their relationships. In addition, the assessment of within-patient changes and clinically relevant QTc thresholds provides a more nuanced interpretation of the observed ECG changes than comparison of group means alone.

Nevertheless, several limitations should be acknowledged. First, the observational, single-center design and the absence of a healthy control group or an RA comparator group not receiving anti-TNF therapy, limit causal inference and preclude attribution of the observed changes specifically to TNF inhibition. The parallel improvements in inflammatory markers, disease activity, and ECG-derived repolarization indices may therefore reflect the broader effects of reduced systemic inflammatory burden rather than a direct electrophysiological effect of anti-TNF therapy. Second, although percentage-based intraobserver and interobserver variability was assessed during the original ECG evaluation, formal ICC-based reliability estimates with 95% confidence intervals could not be calculated because individual repeated observer-level measurements were not available. Third, serial echocardiographic assessment, ambulatory rhythm monitoring, cardiac magnetic resonance imaging, and follow-up NT-proBNP measurements were not available. Moreover, arrhythmias, cardiovascular events, cardiovascular hospitalizations, and sudden cardiac events were not systematically assessed during the 24-week follow-up; therefore, the observed changes in repolarization indices cannot be interpreted as evidence of reduced clinical arrhythmic or cardiovascular risk. Fourth, the study was not designed or adequately powered for comparisons among individual TNF inhibitors. Potential differences among adalimumab, etanercept, and golimumab therefore cannot be excluded, and the findings should be interpreted at the anti-TNF class level. Fifth, BMI and smoking status were not systematically available, preventing assessment of these potentially relevant cardiovascular confounders. Finally, the 24-week follow-up period did not allow assessment of the long-term clinical or prognostic significance of the observed ECG changes.

## 5. Conclusions

In conclusion, anti-TNF therapy in patients with RA was associated with significant improvements in several ECG-derived ventricular repolarization indices over approximately 24 weeks, occurring alongside marked reductions in inflammatory and disease activity. Cross-sectional associations between Tp-e-related indices and inflammatory/disease burden were observed; however, longitudinal analyses did not demonstrate a consistent relationship between the magnitude of inflammatory improvement and the magnitude of repolarization change. Furthermore, reductions in—QTc were not accompanied by changes in the proportion of patients exceeding clinically relevant QTc thresholds. These findings support an association between systemic inflammatory burden and ventricular repolarization characteristics in RA but should not be interpreted as evidence of a direct antiarrhythmic effect of TNF inhibition. Future prospective multicenter studies incorporating advanced cardiac imaging, ambulatory rhythm monitoring, circulating biomarkers, and long-term cardiovascular outcomes are required to determine whether these electrophysiological improvements translate into clinically meaningful cardiovascular benefit.

## Figures and Tables

**Table 1:** Clinical characteristics, laboratory and findings of the patients.

	Pre-Treatment Anti-TNF Group (*n* = 115)	Post-Treatment Anti-TNF Group (*n* = 115)	*p* Value
**Demographic Parameters**			
Age (mean ± SD), years	54.4 ± 12.2	-	
Female gender, *n* (%)	94 (81.7)	-	
Rheumatoid factor positivity, *n* (%)	95 (82.6)	-	
Anti-CCP positivity, *n* (%)	92 (80)	-	
Disease duration (mean ± SD), months	72 ± 11.2	-	
**Laboratory parameters**			
C-reactive protein (mg/L)	23.2 ± 7.4	2.3 ± 1.3	**<0.001**
Erythrocyte sedimentation rate (mm/h)	42.1 ± 8.3	11.3 ± 3.4	**<0.001**
Systolic blood pressure (mmHg)	116.2 ± 11.4	117.0 ± 10.1	0.787
Diastolic blood pressure (mmHg)	74.7 ± 7.5	74.3 ± 7.2	0.871
Disease Activity Score-28	5.9 ± 0.1	2.4 ± 0.3	**<0.001**
HAQ score	1.3 ± 0.2	0.3 ± 0.07	**<0.001**
Creatinine (mg/dL)	0.72 ± 0.17	0.71 ± 0.12	0.657
WBC (10^3^/mL)	7.9 ± 1.7	6.5 ± 1.5	**<0.001**
Hemoglobin (g/dL)	12.1 ± 1.7	12.9 ± 0.9	**<0.001**
Platelet (10^3^/mL)	258.7 ± 64	252.4 ± 57.1	**<0.001**
Lymphocyte (10^3^/mL)	1.8 ± 0.7	1.6 ± 0.4	**0.007**
Neutrophil (10^3^/mL)	5.8 ± 1.8	3.7 ± 1.4	**<0.001**
**Anti-TNF agent, *n* (%)**			
Adalimumab	60 (52.2)	60 (52.2)	-
Etanercept	30 (26.1)	30 (26.1)	-
Golimumab	25 (21.7)	25 (21.7)	-

CCP: cyclic citrullinated peptide; HAQ: Health Assessment Questionnaire; WBC: White blood cell.

**Table 2:** Electrocardiographic findings of the study population.

	Pre-Treatment Anti-TNF	Post-Treatment Anti-TNF (at Week 24)	*p* Value
Heart rate (beats/min)	79.2 ± 11.6	78.8 ± 11.3	0.163
QRS duration (ms)	89.9 ± 9.4	90.0 ± 10.0	0.698
QT interval (ms)	370.3 ± 33.7	367.3 ± 10.0	**<0.001**
QTc interval (ms)	423.2 ± 41.1	419.6 ± 43.3	**<0.001**
Tp-e (ms)	85.1 ± 13.6	75.3 ± 13.5	**<0.001**
cTp-e (ms)	95.0 ± 11.3	85.6 ± 10.2	**<0.001**
Tp-e/QT ratio	0.20 ± 0.04	0.18 ± 0.03	**<0.001**

TNF: Tumor necrosis factor; QTc: corrected QT; Tp-e: transmural dispersion of repolarization; cTp-e: corrected transmural dispersion of repolarization.

**Table 3:** Correlation coefficients: *p* values between laboratory variables Tp-E and Tp-E/QT.

Variables	Tp-e		Tp-e/QT	
	r	*p*	r	*p*
C-reactive protein (mg/L)	0.56	**<0.001**	0.43	**<0.001**
Erythrocyte sedimentation rate (mm/h)	0.57	**<0.001**	0.53	**<0.001**
Disease Activity Score28-CRP	0.29	**0.002**	0.25	**0.007**
HAQ score	0.33	**<0.001**	0.22	**0.018**
Disease duration (months)	0.58	**<0.001**	0.49	**<0.001**

HAQ: Health Assessment Questionnaire; Tp-e: transmural dispersion of repolarization.

**Table 4:** Correlations between changes in inflammatory/disease activity measures and changes in ventricular repolarization indices.

Variables	ΔTp-e		ΔTp-e/QT	
	r	*p*	r	*p*
ΔC-reactive protein (mg/L)	0.014	0.879	0.148	0.115
Δ Erythrocyte sedimentation rate (mm/h)	0.067	0.476	−0.025	0.795
ΔDisease Activity Score28-CRP	0.201	**0.031**	0.099	0.291
ΔHAQ score	0.092	0.329	0.095	0.314

HAQ: Health Assessment Questionnaire; Tp-e: transmural dispersion of repolarization.

## Data Availability

The datasets generated and/or analyzed during the current study are not publicly available due to institutional regulations and the protection of patient confidentiality but are available from the corresponding author on reasonable request, subject to approval by the relevant institutional authorities.
